# Frequency and Outcome of Graft versus Host Disease after Stem Cell Transplantation: A Six-Year Experience from a Tertiary Care Center in Pakistan

**DOI:** 10.1155/2013/232519

**Published:** 2013-06-27

**Authors:** Natasha Ali, Salman Naseem Adil, Mohammad Usman Shaikh, Nehal Masood

**Affiliations:** ^1^Department of Pathology and Microbiology, The Aga Khan University and Hospital, P.O. Box 3500, Stadium Road, Karachi 74800, Pakistan; ^2^Department of Medicine, The Aga Khan University and Hospital, P.O. Box 3500, Stadium Road, Karachi 74800, Pakistan

## Abstract

*Objective*. The objective of this study was to evaluate the frequency and outcome of graft versus host disease after stem cell transplantation for various haematological disorders in Pakistan. *Materials and Methods*. Pretransplant workup of the patient and donor was performed. Mobilization was done with G-CSF 300 **μ**g twice daily for five day. Standard GvHD prophylaxis was done with methotrexate 15 mg/m^2^ on day +1 followed by 10 mg/m^2^ on days +3 and +6 and cyclosporine. Grading was done according to the Glucksberg classification. *Results*. A total of 153 transplants were done from April 2004 to December 2011. Out of these were allogeneic transplants. There were females and males. The overall frequency of any degree of graft versus host disease was 34%. Acute GvHD was present in patients while had chronic GvHD. Grade II GvHD was present in patients while grade III and IV GvHD was seen in patients each. Acute myeloid leukemia and chronic myeloid leukemia were most commonly associated with GvHD. The mortality in acute and chronic GvHD was 8.8% and 12% respectively. *Conclusion*. The frequency of graft versus host disease in this study was 34% which is lower compared to international literature. The decreased incidence can be attributed to reduced diversity of histocompatibility antigens in our population.

## 1. Introduction

Allogeneic haemopoietic stem cell transplant is an established treatment modality for many malignant and non-malignant conditions [1]. Its use over the last decade has extensively expanded which includes nonmyeloablative transplant, donor lymphocyte infusions, and umbilical cord blood transplant [2, 3]. As the numbers of procedures continue to increase with 25,000 transplants being performed annually, the survival benefit, however, is complicated by graft versus host disease (GvHD) leading to significant morbidity, mortality, and limitation of its usage [4]. Most of the laboratories in the world have adopted the high-resolution testing modality for human leukocyte antigen (HLA) typing. Every conditioning protocol incorporates the use of immunosuppression most commonly with cyclosporine and methotrexate. Despite these measures, GvHD remains an important cause of transplant-related mortality and morbidity leading to limitation of its usage [5].

Approximately 30 years ago, the prerequisites of acute GvHD were described by Billingham. These included immunologically competent cells in sufficient numbers to be present in the graft. The host to possess transplant isoantigens not present in the graft and its immune system should be incapable of mounting a reaction against the graft [6]. These immunologically competent T cells can cause GvHD in various clinical scenarios when they are transfused from blood products and solid organs to recipients who are unable to mount an immune response against these cells. In the early 1990s, based on Seattle experience, GvHD was defined depending on the time at which it occurred; that is, early was defined as occurring before 100 days and chronic occurring after the defined period [7]. In 2005, the National Institutes of Health Consensus included an entity of late onset acute GvHD (occurring after day 100) revealing features of both acute and chronic GvHD [8].

Despite the 10/10 HLA antigen match using high-resolution typing, approximately 30% of recipients of allograft develop acute GvHD [9]. In 1990, Martin et al. described acute graft versus host disease as involvement of skin which is the most frequent organ involved (in 81%), gastrointestinal tract in 54%, and liver in 50% of the patients [10]. The extent of involvement of these organs determines the severity of acute GvHD. Overall grades are I (mild), II (moderate), III (severe), and IV (very severe). The overall survival for grades III and IV is very poor with 25% and 5% survival rates, respectively [11]. 

Chronic GvHD is one of the main causes of morbidity and mortality after stem cell transplant [12]. In Recipients who have received 10/10 HLA-matched allografts, the incidence has been estimated to range from 30 to 50% in long-term survivors [13]. This incidence proportionately increases with HLA disparity. Previously, chronic GvHD was classified as “limited” versus “extensive”, and again in 2005, the new staging system considered the number of organs involved along with functional impairment [8]. Organ-specific scores were assigned and overall stage was established as follows: (1) mild chronic GvHD involves one or two sites (except the lungs, which results in a classification of moderate chronic GVHD at a minimum), with no clinically significant functional impairment (maximum score of 2 in all affected sites); (2) moderate chronic GVHD involves at least one organ/site with clinically significant involvement but no major functional disability (maximum score of 2 in any site) orthree or more organs or sites with no clinically significant functional impairment (max score of 1 in all organs/sites); (3) severe chronic GVHD indicates major disability (score of 3 in any organ/site) [14].

Graft versus host disease prophylaxis has been incorporated in every conditioning treatment protocol and it includes cyclosporine on day 1 with serial monitoring of levels along with administration of three doses of methotrexate [15].

In a developing country like Pakistan, stem cell transplant is being performed in three centers throughout the country. Our center was established in 2004. In this paper we present the frequency and outcome of graft versus host disease in allogeneic stem cell transplant over a period of six years at our center. We have evaluated the distribution of haematological disorders most commonly associated with graft versus host disease; the outcome of patients and the comparative differences in these variables between our population and international literature have been elaborated. 

## 2. Materials and Methods

All patients with nonmalignant and malignant haematological disorders with HLA-matched donors were selected for the procedure.

### 2.1. Pretransplant Workup

Complete blood counts, liver and kidney function tests, and infectious disease profile (consisting of hepatitis B surface antigen, hepatitis C antibody, HIV antibody, cytomegalovirus, Mantoux test, and chest X ray) along with blood grouping and coagulation testing were performed in all donors. For patients, screening included all the aforementioned investigations along with pulmonary function tests, echocardiography, and dental evaluation.

### 2.2. Stem Cell Mobilization

All donors were given granulocyte-colony stimulating factor (G-CSF) at a dose of 5 *μ*g/kg twice daily for five days prior to harvest. Patients with donors less than five years received bone marrow only as the stem cell source. In patients with aplastic anaemia, peripheral blood and bone marrow stem cells were the preferred source. In all other conditions, peripheral blood progenitor cells only were used as the source of stem cells.

### 2.3. Conditioning Regimen

Patients with thalassemia, acute myeloid leukemia, chronic myeloid leukemia, biphenotypic leukemia, and Philadelphia-negative, acute lymphoblastic leukemia received Busulfan (1 mg/kg/day q6 hours for four days) and cyclophosphamide (60 mg/kg/day for two days) as conditioning chemotherapy. Class III thalassemic patients received conditioning with hyperchelation protocol [16]. Total body irradiation (1.5cGY x twice a day for four days) and Cyclophosphamide (60 mg/kg/day for two days) were used in patients with Philadelphia-positive acute lymphoblastic leukemia and those with one-antigen mismatch donors (*n* = 6).

In aplastic anaemia, antithymocyte globulin (10 mg/kg/day for three days) and Cyclophosphamide (50 mg/kg/day for four days) were used. Patients with Fanconi's anaemia received conditioning with fludarabine (30 mg/kg/day for three days).

### 2.4. Infectious Disease Prophylaxis

Patients were admitted in protective isolation equipped with HEPA filter, positive pressure, and laminar airflow ventilation. Standard prophylaxis with ciprofloxacin (500 mg twice daily or 20–30 mg/kg/two divided doses), fluconazole (200 mg once daily or 6 mg/kg/day), and valaciclovir (500 mg twice daily or 10 mg/kg/twice daily) was started in all patients on day 5. All patients were provided with neutropenic diet.

### 2.5. Graft versus Host Disease Prophylaxis

Intravenous Cyclosporine was started on day 1 and doses were adjusted according to drug levels. Optimum adult range was 200–250 ng/dL. For paediatric patients, levels were maintained between 150 and 200 ng/dL. Methotrexate 15 mg/m^2^ was administered on day +1, while 10 mg/m^2^ was given on days +3 and +6. Irradiated and leukocyte-reduced blood products were used throughout admission as well as in the posttransplant period. Clinical grading for acute GVHD was adapted from Przepiorka et al. [17]. For chronic GVHD, criteria defined by Shulman et al. were used [7].

### 2.6. Statistical Analysis

All data was entered on SPSS version 19 (SPSS Inc., Chicago, IL, USA) for computing means, standard deviation, and range of all descriptive variables.

## 3. Results

A total of stem cell transplants were performed from April 2004 to December 2011. Out of these *n* = 101 were allogeneic transplant procedures and were autologous. Allogeneic transplants were done for aplastic anaemia (*n* = 36), thalassemia major (*n* = 21), chronic myeloid leukemia (*n* = 11), acute myeloid leukemia (*n* = 10), acute lymphoblastic leukemia (*n* = 8), myelodysplastic syndrome (*n* = 6), Fanconi's anaemia (*n* = 5), and *n* = 1 each for haemophagocytic lymphohistiocytosis, osteopetrosis, biphenotypic leukemia and mantle cell lymphoma. There were 72 males and 29 females. The median age ± SD was 18 ± 12.4 years (range: 2–54 years). Four patients received stem cells from HLA-matched parents. Five patients received stem cells from one HLA antigen mismatch sibling donors. One patient received stem cells from one HLA antigen mismatch parent. Approximately 30% (*n* = 32) of the transplants were gender mismatched. There were 7 male patients who received stem cells from female donors previously sensitized by pregnancy.

**Figure 1:**
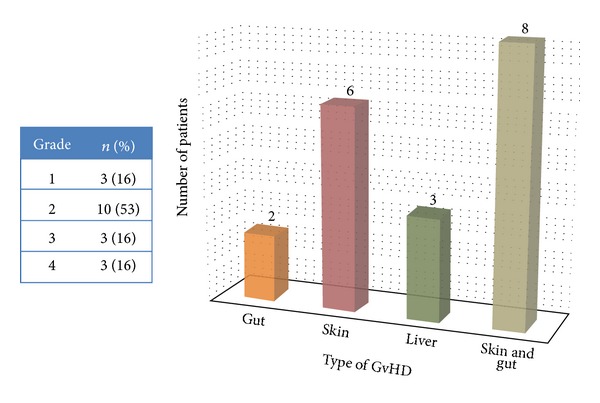
Acute GvHD grade and organ of involvement (*n* = 19/34).

**Figure 2:**
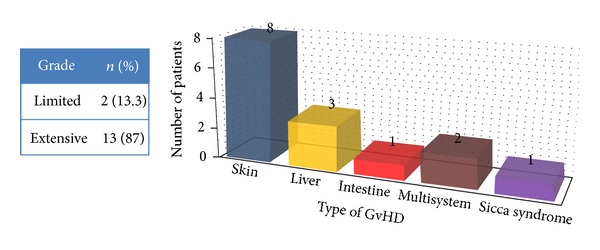
Chronic GvHD grade and organ of involvement (*n* = 15/34).

The overall frequency of graft versus host disease was 34% (*n* = 34). Acute GvHD was present in 19 patients while 15 had chronic GvHD. According to age groups, 7 pediatric patients developed GvHD while 27 were adults (Table 1). The mean mononuclear stem cell count (MNC) was 7.7 × 10^8^/kg, and the mean CD34^+^ stem cell dose was 5.5 × 10^6^/kg. Grade II acute GvHD was present in 10 patients (52.6%), while grade III and, IV was seen in 3 patients each. The most common site of involvement in acute GvHD was skin and gut (*n* = 8) followed by skin only in 6 patients (Figure 1). Out of 15 patients with chronic GvHD, 13 had extensive involvement (Figure 2). The most common site of involvement was skin (*n* = 8) followed by liver (*n* = 3).

The most common haematological disorders associated with GvHD were acute myeloid leukemia (70%) followed by chronic myeloid leukemia (63%) and acute lymphoblastic leukemia (50%); see Table 2. Out of 17 patients who received “bone marrow only” as a source of stem cells, none of them developed acute or chronic GvHD. For patients who received peripheral blood only, out of 52, 15 (29%) developed acute GvHD whereas 14 (27%) developed chronic GvHD. There were 32 patients who received peripheral blood and bone marrow as a combined source of stem cells. The frequency of acute GvHD in this group was 12.5% (*n* = 4) and that of chronic GvHD was 3.1% (*n* = 1).

Biopsy was performed in 32/34 patients and GvHD was diagnosed in 84% of the cases. Death due to acute and chronic GvHD was seen in 3 (8.8%) and 4 (12%), respectively.

## 4. Discussion

Development of graft versus host disease is generally considered to harbor the beneficial effect of graft versus leukemia effect. However, in majority of patients it remains to be the single determinant of long-term survival and quality of life after bone marrow transplant. Despite contemporary prophylaxis with cyclosporine, methotrexate, and irradiated blood products, GvHD develops in 50% of the patients receiving allografts. Depletion of  T cells from donor can result in controlling GvHD but in turn may lead to increased rates of graft failure, impaired immune reconstitution, infections, relapse, and posttransplant lymphoproliferative disorder [18, 19]. Apart from genetic factors, other causes of eliciting a graft versus host response include donor type, haemopoietic stem cell dose, and multiparous female donors [20]. In our study, all seven male patients who received stem cells from multiparous donors developed GvHD. In 2011, Flowers et al. [21] reported an increased risk of grade 2–4 acute GvHD when a female donor was used in a male recipient. Similar results in children have been reported by Kondo et al. in 2011 [22]. One of the male patients in our study was three years old with thalassemia major who received peripheral blood stem cells from his mother and developed chronic extensive GvHD.

In recipients of complete match sibling allografts, the incidence of acute GvHD ranges from 35% to 45%. This incidence increases with the amount of HLA disparity and has been reported as high as 60% to 80% in recipients of one antigen mismatch allografts [23, 24]. In our study, the incidence of acute graft versus host disease was approximately 19%. This frequency is relatively lower when compared to local data and studies done within the region by Hashmi et al. [25] and Ghavamzadeh et al. [26], respectively. Furthermore, acute GvHD was more frequently seen in adults as compared to pediatric patients in our study. The incidence has been reported as 60.2% by Liu et al. [27], and 29% by Shaw et al. [28] and our frequency is lower to the figure quoted in both studies done in pediatric cohort. With the rise in number of allogeneic stem cell transplants, advances in pretransplant conditioning regimens, and posttransplant care, the incidence of chronic GvHD has also increased in long-term survivors. In recipients of HLA-matched sibling donors, the incidence of chronic GvHD has been reported as 30%–50%. Sorror et al. [29] have reported cumulative incidence of 42% at 2 years in patients with advanced haematological malignancies. Cantu-Rodriguez et al. reported an incidence of 29.9% [30]. Both of these figures are in sharp contrast to the frequency seen in our patients which was approximately 15%. Our cohort mainly consisted of benign haematological disorders like aplastic anaemia and beta thalassemia major which prompted us to use either bone marrow only or bone marrow and peripheral blood as a source of stem cells. This could be one of the reasons behind the lower frequency in our study since none of the patients whose stem cells were from bone marrow developed GvHD. Secondly, since graft versus host disease after stem cell transplant between siblings matched for major histocompatibility complex develops presumably as a result of differences in the minor histocompatibility antigens between the donor and recipients, hypothetically this may mean that the degree of HLA polymorphism is lower in Pakistani population when compared with western cohort.

A graft versus leukemia effect can be obtained by transfusion-induced suppression of host's hematopoiesis resulting from sharing of histocompatibility antigens with the leukemia. In order to achieve this effect, we used growth factor-stimulated peripheral blood only as a source of stem cells in acute and chronic leukemia. Due to this, the most common haematological disorders associated with graft versus host disease were acute and chronic leukemia. A meta-analysis done by Chang et al. [31] also identified the aforementioned three most common haematological disorders associated with GvHD. Recently, peripheral blood stem cells have gained acceptance they and in 71% of allogeneic transplants, are being used as stem cell source. Several randomized studies have reported faster hematopoietic and immune recovery. A retrospective study in 329 patients comparing bone marrow and peripheral blood stem cells showed a cumulative acute GvHD incidence of 51% and 54% in the bone marrow and peripheral blood stem cells (PRBSCT) group, respectively [32]. The incidence of chronic GvHD was 48% in patients who received PRBSCT. Although our incidence is much lower from this cohort, interestingly, none of the patients in group who received bone marrow only stem cells went on to develop acute or chronic GvHD. Our center currently does not use T-cell-depleted PBSCT. This could be one of the reasons for the striking difference in our results of GvHD between the two stem cell sources.

In patients with acute GvHD, the transplant-related mortality significantly increased and it correlated with the grade and organ of involvement. The EBMT group has reported a mortality incidence of 25% in patients with GvHD [33]. In our study, mortality due to acute and chronic GvHD was 9% and 12%, respectively, which is much lower than that quoted in the international literature. Reasons could be due to younger age group of patients receiving allografts from young sibling donors and low occurrence of grade III-IV GvHD eventually leading to much lower frequency of chronic GvHD.

## 5. Conclusion

The incidence of acute and chronic GvHD in this study was lower as compared to the international literature. Overall frequency was 34%. Mortality due to acute and chronic GvHD was 8.8% and 12%, respectively. GvHD developed mainly in patients receiving peripheral blood stem cells. The decreased incidence of GvHD can be due to reduced disparity of histocompatibility antigens in our population. 

## Figures and Tables

**Table 1:** Frequency of GvHD according to age groups.

Age group	Total (*n*)	GvHD (*n*)	
		Yes	No
Pediatric	44	7	37
Adult	57	27	30

Total	101	34	67

**Table 2:** Frequency of GvHD according to haematological diseases.

Diagnosis	GvHD *n* (%)	Total number of cases
*β*-Thalassemia major	5	21
Aplastic anaemia	5	36
Acute myeloid leukemia	7	10
Acute lymphoblastic leukemia	4	08
Chronic myeloid leukemia	7	11
Myelodysplastic syndrome	4	06
Biphenotypic leukemia	1	01
Osteopetrosis	1	01
HLH	0	01
Fanconi's anaemia	0	05
Mantle cell lymphoma	0	01

Total	34	101
